# Site dependence of the magnetocaloric effect in Mn_5−*x*_Fe_*x*_Si_3_

**DOI:** 10.1107/S1600576722007440

**Published:** 2022-09-06

**Authors:** Mohammed Ait Haddouch, Nour Abboushi, Neetika Sharma, Andreas Eich, Andrzej Grzechnik, Cheng Li, Martin Tolkiehn, Husain Alsamamra, Jörg Voigt, Karen Friese

**Affiliations:** a Jülich Centre for Neutron Science-2, Forschungszentrum Jülich GmbH, 52425 Jülich, Germany; b Lehrstuhl für Experimentalphysik IVc, RWTH Aachen University, 52056 Aachen, Germany; c Physics Department, Al-Quds University, 90612 Abu Dis, State of Palestine; d Institute for Crystallography, RWTH Aachen University, Jägerstraße 17–19, 52066 Aachen, Germany; e Oak Ridge National Laboratory, Oak Ridge, Tennessee, USA; f Photon Science, Deutsches Elektronen-Synchrotron (DESY), Hamburg, Germany; HPSTAR and Harbin Institute of Technology, People’s Republic of China

**Keywords:** magnetocaloric effect, magnetic structure, neutron diffraction, synchrotron diffraction, site dependence

## Abstract

The nuclear and magnetic structures of Mn_3_Fe_2_Si_3_ are investigated in the temperature range from 20 to 300 K. The magnetic properties of Mn_3_Fe_2_Si_3_ were measured on a single crystal. The compound undergoes a paramagnetic to antiferromagnetic transition at *T*_N2_ ≃ 120 K and an antiferromagnetic to antiferromagnetic transition at *T*_N1_ ≃ 69 K. A similar sequence of magnetic phase transitions is found for the parent compound Mn_5_Si_3_ upon temperature variation, but the field-driven transition observed in Mn_5_Si_3_ is not found in Mn_3_Fe_2_Si_3_, resulting in a strongly reduced magnetocaloric effect. Structurally, the hexagonal symmetry found for both compounds under ambient conditions is preserved in Mn_3_Fe_2_Si_3_ through both magnetic transitions, indicating that the crystal structure is only weakly affected by the magnetic phase transition, in contrast to Mn_5_Si_3_ where both transitions distort the nuclear structure. Both compounds feature a collinear high-temperature magnetic phase AF2 and transfer into a non-collinear phase AF1 at low temperature. While one of the distinct crystallographic sites remains disordered in the AF2 phase in the parent compound, the magnetic structure in the AF2 phase involves all magnetic atoms in Mn_3_Fe_2_Si_3_. These observations imply that the distinct sites occupied by the magnetic atoms play an important role in the magnetocaloric behaviour of the family.

## Abstract 2

The nuclear and magnetic structures of Mn_3_Fe_2_Si_3_ are determined and the magnetic properties are compared with those of the parent compound Mn_5_Si_3_. The results imply that the distinct magnetic sites play an important role in the magnetocaloric behaviour of the family.

## 1. Introduction

Magnetocaloric cooling has become one of the most promising candidates for energy-efficient and environmentally friendly refrigeration and can potentially replace the conventional vapour-compression-based technologies (Yu *et al.*, 2003 ▸; Brück, 2005 ▸; Gschneidner & Pecharsky, 2008 ▸; Takeuchi & Sandeman, 2015 ▸). The magnetocaloric refrigeration cycle is based on materials which exhibit the magnetocaloric effect (MCE), that is, a change of temperature upon adiabatic magnetization/demagnetization (Pecharsky & Gscheidner, 1999 ▸).

**Figure 1:**
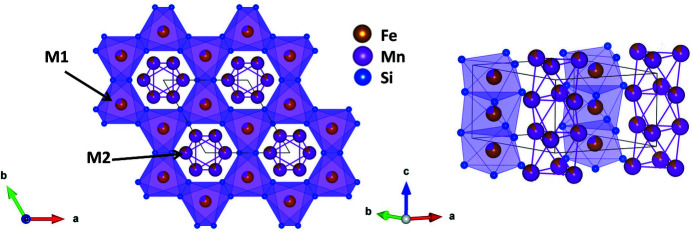
(Left) A projection of the crystal structure of Mn_3_Fe_2_Si_3_ (*P*6_3_/*mcm*) at 300 K along the [001] direction and (right) a projection approximately along the [120] direction.

The series of compounds in the system Mn_5−*x*_Fe_*x*_Si_3_ are well known for their magnetocaloric properties (Songlin *et al.*, 2002 ▸; Gourdon *et al.*, 2014 ▸; Hering *et al.*, 2015 ▸; Maraytta *et al.*, 2019 ▸; Singh *et al.*, 2020 ▸). The crystal structure of the parent compound Mn_5_Si_3_ can be described in space group *P*6_3_/*mcm* (Bińczycka *et al.*, 1973 ▸). Within the crystal structure two non-equivalent sites are available for the paramagnetic atoms. The *M*1 site is surrounded by Si atoms in the form of a distorted octahedron, which shares common faces with its analogues, forming infinite chains along the *c* axis. The atoms on the *M*2 site form empty [□(*M*2)_6_] octahedra, which also form infinite chains of face-shared octahedra along the **c** direction. Neighbouring _∞_[(*M*1)Si_3_] chains share common edges with each other and form channels which are occupied by chains of empty octahedra of composition _∞_[□(*M*2)_3_] (Fig. 1 ▸). It is well known that Fe atoms preferentially occupy the *M*1 site and Mn atoms are preferentially incorporated onto the *M*2 site (Songlin *et al.*, 2002 ▸; Hering *et al.*, 2015 ▸).

The compounds of this series are ideal for studying the underlying mechanism of the magnetocaloric effect for various reasons:

(i) The nature of the magnetic ordering changes depending on the composition. While for small values of *x* antiferromagnetic structures are reported, ferromagnetism is observed for *x* > 3 (Narasimhan *et al.*, 1970 ▸; Songlin *et al.*, 2002 ▸; Vinokurova *et al.*, 1995 ▸; Hering *et al.*, 2015 ▸; Singh *et al.*, 2020 ▸).

(ii) Compounds with an Fe content *x* ≥ 3 show a direct MCE (the magnetic entropy decreases with the application of the magnetic field and the material heats up), while the parent compound Mn_5_Si_3_ shows an inverse MCE, *i.e.* the magnetic entropy rises with the application of the field and the material cools down. The inverse MCE is observed at the transition from a collinearly ordered arrangement of the spins to a non-coplanar arrangement (Gottschilch *et al.*, 2012 ▸; Biniskos *et al.*, 2018 ▸; Luccas *et al.*, 2019 ▸), while in the compound with *x* = 4 a direct MCE is observed at the paramagnetic to ferromagnetic transition (Gourdon *et al.*, 2014 ▸; Hering *et al.*, 2015 ▸; Maraytta *et al.*, 2019 ▸).

(iii) The availability of two significantly different sites for the paramagnetic atoms within the structure gives the opportunity to elucidate the role of magnetic atoms occupying multiple sites in magnetocaloric materials.

(iv) In addition, large single crystals of the compounds up to 10 cm in length and 1–2 cm diameter are readily available. This is of advantage when it comes to elucidating the underlying crystal and magnetic structures, the magnetic anisotropy, or the spin and/or lattice dynamics (Biniskos *et al.*, 2017 ▸, 2018 ▸; Maraytta *et al.*, 2020 ▸).

The compound investigated here, Mn_3_Fe_2_Si_3_, is special within the series, as the stoichiometry would allow perfect site order, *i.e.* all *M*1 sites occupied by Fe atoms and all *M*2 sites occupied by Mn. Like the parent compound Mn_5_Si_3_, Mn_3_Fe_2_Si_3_ is known to have two different antiferromagnetic phases, yet their magnetic structures are still unknown.

In this work we study the details of the crystal structure of Mn_3_Fe_2_Si_3_ as a function of temperature using high-resolution synchrotron single-crystal diffraction and determine for the first time the magnetic structure of Mn_3_Fe_2_Si_3_ in the AF1 and AF2 phases using neutron powder diffraction. We compare the direction-dependent macroscopic magnetic and magneto­caloric properties of single-crystalline Mn_3_Fe_2_Si_3_ and the parent compound Mn_5_Si_3_ and discuss the differences in the light of the structural investigations.

## 2. Experimental procedures

### 2.1. Sample preparation

Single crystals of Mn_5_Si_3_ and Mn_3_Fe_2_Si_3_ were obtained according to the method described by Hering *et al.* (2015 ▸). Chemical analysis using inductively coupled plasma with optical emission spectroscopy showed no deviations from the ideal stoichiometry (see Table S1 in the supporting information).

### 2.2. Magnetization

Fragments of the oriented single crystal with a mass of 6–12 mg were cut using spark erosion. Measurements of the magnetization parallel and perpendicular to the hexagonal [001] direction with varying temperature and magnetic field were carried out in the temperature region between 5 and 380 K and magnetic fields in the range −9 T ≤ μ_0_ *H* ≤ 9 T using the vibrating sample magnetometer (VSM) option of a PPMS and PPMS Dynacool from Quantum Design, respectively. Isothermal measurements were performed to identify the magnetic phases for the different directions of the applied field and to determine the magnetic entropy change Δ*S*_iso_. As the magnetization curves became featureless for **H** ∥ [001] for *T* > *T*_N1_, isotherms were only recorded up to 73 K for this field direction. In the case of Mn_5_Si_3_, the field-dependent magnetization was measured in sweep mode, *i.e.* the field was varied at a rate of 5 mT s^−1^ and the magnetization was recorded continuously. For Mn_3_Fe_2_Si_3_, preliminary measurements had shown that the measured magnetization changes with the thermomagnetic history. Therefore, we employed the following temperature protocol: The samples were initially cooled to 140 K. Subsequently a field of 9 T was applied and the sample was cooled to the target temperature at a rate of 5 K min^−1^, and then a field loop between 9 T and −9 T was recorded. At the end of the loop, the sample was again heated to 140 K and cooled to the next target temperature to ensure as identical starting conditions for the magnetization loop as possible.

### 2.3. Neutron powder diffraction

Time-of-flight neutron powder diffraction data on about 5 g of Mn_3_Fe_2_Si_3_ powder were recorded using the POWGEN diffractometer at Oak Ridge National Laboratory (Huq *et al.*, 2011 ▸) at temperatures of 20, 50, 90, 105 and 300 K. Data were recorded using two different bands, one with central wavelength CWL = 0.8 Å (*d* spacing coverage 0.1340–8.200 Å) and the other with CWL = 2.665 Å (1.0701–22.9342 Å).

### 2.4. Synchrotron single-crystal diffraction

Synchrotron single-crystal diffraction data were measured on beamline P24 of PETRA III at DESY (Hamburg, Germany) at a wavelength of λ = 0.44279 Å using a MarCCD165 detector. Data sets were collected upon cooling (*T* = 300, 250, 200, 150, 125, 100, 80, 60, 40 and 20 K) using an open-flow helium cryostat. Data were reduced with the *CrysAlisPro* software (Rigaku Oxford Diffraction, 2015 ▸). All refinements of the powder and single-crystal data of Mn_3_Fe_2_Si_3_ were performed with the program *JANA2006 ▸* (Petříček *et al.*, 2014 ▸).

## 3. Results

### 3.1. Magnetization measurements

**Figure 2:**
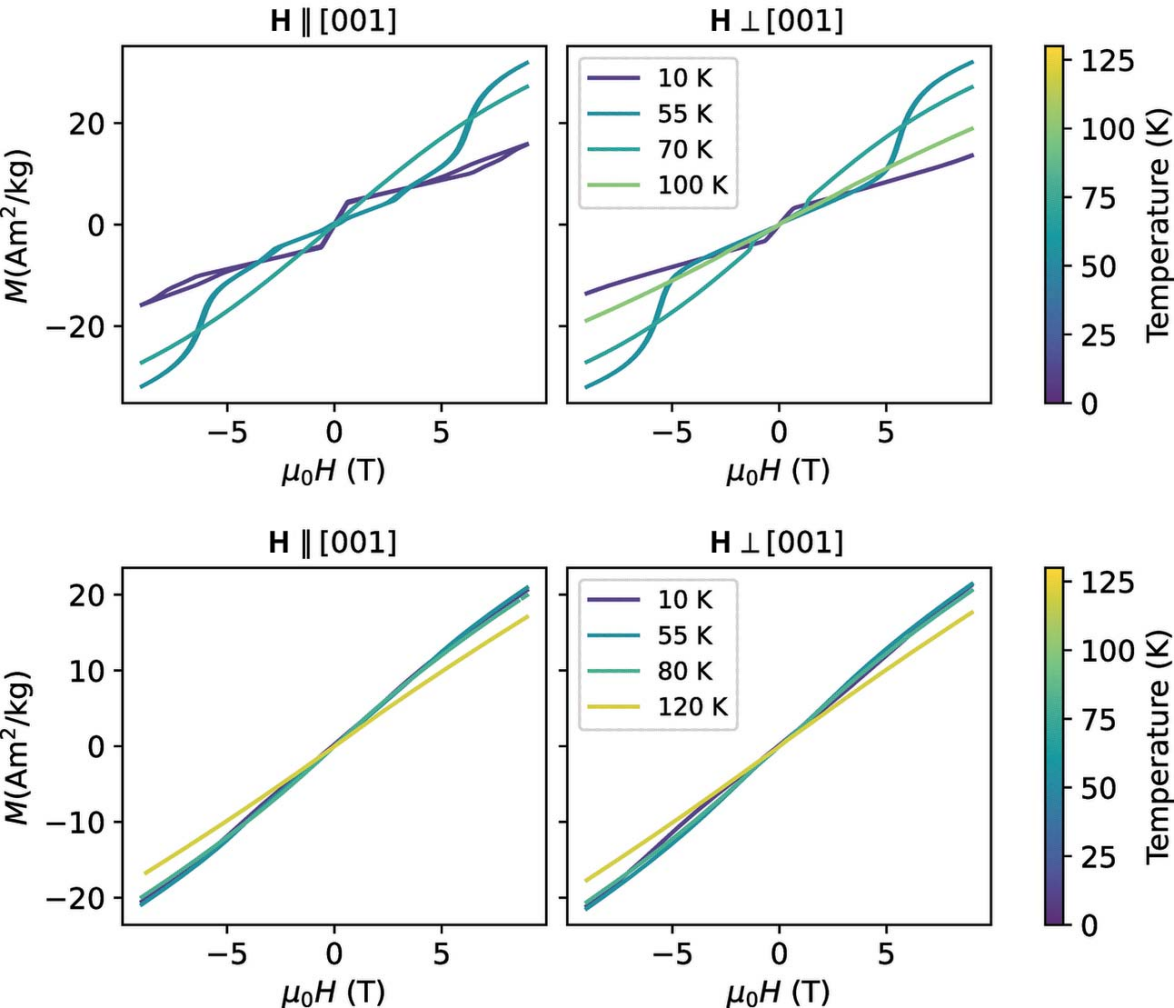
Isothermal magnetization data: full hysteresis loops at selected temperatures in AF1 and AF2 for the field direction (left) ∥ and (right) ⊥ [001] for (top) Mn_5_Si_3_ and (bottom) Mn_3_Fe_2_Si_2_. The line colour indicates the temperature of the isothermal measurement.

**Figure 3:**
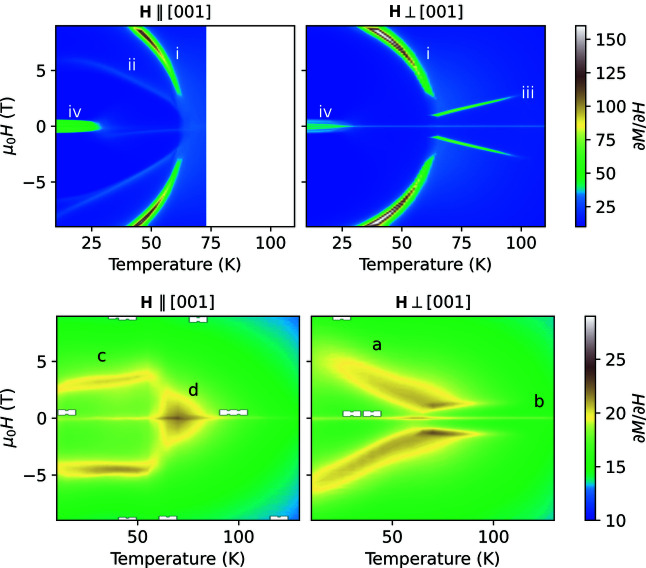
False-colour plots of ∂*M*/∂*H* interpolated from the isothermal magnetization curves upon a field change from 9 T to −9 T for (top) Mn_5_Si_3_ measured with 3 K temperature steps and (bottom) Mn_3_Fe_2_Si_3_ measured with 5 K temperature steps with field (left) ∥ and (right) ⊥ [001]. Note the different colour ranges of the top and bottom panels. Labels annotate the different features as discussed in the text.

Isothermal magnetization measurements at selected temperatures in AF1, AF2 and the paramagnetic state (Fig. 2 ▸) for Mn_5_Si_3_ and Mn_3_Fe_2_Si_3_ for a field applied perpendicular to [001] and parallel to [001] exhibit a plethora of features, which are only visible as small kinks. The field derivatives ∂*M*/∂*H* emphasize the slope changes and visualize the temperature dependence of the different features (Fig. 3 ▸).

In Mn_5_Si_3_, the features labelled (i) and (ii) have been reported in the various magnetization studies on single crystals and powders (Sürgers *et al.*, 2017 ▸; Vinokurova *et al.*, 1990 ▸; Al-Kanani & Booth, 1995 ▸; Songlin *et al.*, 2002 ▸; Das *et al.*, 2019 ▸) and are related to the field-driven transition from the field-induced antiferromagnetic phase AF1* to AF2 and from AF1 to AF1*. In contrast to the results obtained by Sürgers and co-workers, we observe the feature related to the transition from AF1 to AF1* only if the field is applied parallel to the orthorhombic [001] direction.^1^ The splitting that is observed for feature (ii) below 25 K is hysteretic, *i.e.* when the field is varied from −9 T to 9 T it splits at approximately 6 T. In the vicinity of *T*_N1_ ≃ 66 K we observe a slightly elevated ∂*M*/∂*H* for the field applied parallel to [001] and the formation of two sharp kinks labelled (iii) for **H** ⊥ [001]. Below ∼30 K, we observe for both field directions a nearly constant ∂*M*/∂*H* for |μ_0_ *H*| < 0.6 T, labelled (iv). This feature has already been observed in the literature but has not been discussed in detail (Sürgers *et al.*, 2017 ▸; Vinokurova *et al.*, 1990 ▸; Al-Kanani & Booth, 1995 ▸; Songlin *et al.*, 2002 ▸; Das *et al.*, 2019 ▸). It resembles the magnetization of a soft ferromagnet that has not been corrected for the demagnetization field. Recently it has been proposed that the linear increase in the magnetization can be explained as a longitudinal variation of the moment on the *M*1 site (Biniskos *et al.*, 2022 ▸), which explains the behaviour observed here. Feature (iv) narrows down above 30 K for both field directions. For a field parallel to [001] it becomes an open hysteresis loop, which is indicated by the small peak at positive field for increasing field and at negative field for decreasing fields (Fig. S1 in the supporting information). This feature disappears for the field parallel to [001] at *T*_N1_ but persists even beyond *T*_N2_ for the perpendicular direction.

Mn_3_Fe_2_Si_3_ exhibits a much lower slope ∂*M*/∂*H* (compare the range of the colour bars on the right-hand side of the panels). In the low-temperature phase AF1 (*T* < *T*_N1_ = 69 K), we observe the features labelled (c) and (a) for the field parallel and perpendicular to the [001] direction. For both field directions we find only one feature when varying the field at constant temperature, in agreement with pulsed field measurements on powders (Songlin *et al.*, 2002 ▸). Similarly to the results for Mn_5_Si_3_ we observe an enhanced slope around zero field just above *T*_N1_ labelled (d) and two narrow kinks at finite field, which disappear just at *T*_N2_ ≃ 120 K, labelled (b). For this composition, the slope ∂*M*/∂*H* is enhanced for very small fields above *T*_N2_ for both field directions.

From the magnetization data we calculate the isothermal entropy change Δ*S*_iso_ employing the Maxwell relation: 

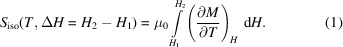

**Figure 4:**
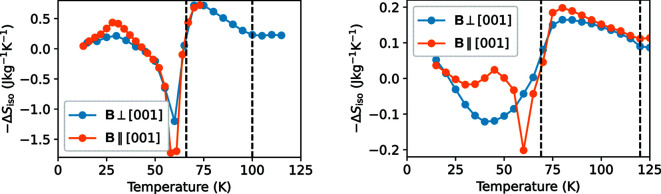
Magnetic entropy changes for a field change Δ*B* = 5 T from isothermal magnetization measurements for (left) Mn_5_Si_3_ and (right) Mn_3_Fe_2_Si_3_. Note that the MCE is underestimated due to the coarse temperature steps of 3 and 5 K, respectively. Dashed vertical lines indicate the transition temperatures *T*_N1_ and *T*_N2_.

In Mn_5_Si_3_, the strong increase in magnetization when entering the AF2 phase from the AF1 phase results in the rather strong inverse MCE (Fig. 4 ▸). The resulting *S*_iso_ varies only slightly for the different directions and is in agreement with the powder results obtained by Songlin *et al.* (2002 ▸). The observed small direction dependence can basically be attributed to the different temperature steps chosen in the measurement for Mn_5_Si_3_.

In comparison, the isothermal entropy change Δ*S*_iso_ in Mn_3_Fe_2_Si_3_ is nearly a factor of 10 smaller. Here a clear direction dependence of the MCE is visible. If the field is applied ∥ [001], an inverse MCE is observed in a narrow temperature region around 60 K. For the direction ⊥ [001], −Δ*S*_iso_ is more negative upon heating in the temperature region between 20 and 55 K and then turns positive above approximately 70 K. Within the AF2 phase, the MCE does not differ strongly for the different field directions.

### 3.2. Crystal and magnetic structure of Mn_3_Fe_2_Si_3_

#### 3.2.1. Refinement of the nuclear structure

In the refinement of the neutron powder data, a pseudo-Voigt profile function and a background described by ten terms of Legendre polynomials combined with 70 manually assigned background points were used. An absorption correction according to Larson & Von Dreele (2004 ▸) and a correction for preferred orientation according to March–Dollase were applied (Larson & Von Dreele, 2004 ▸). Coordinates and displacement parameters of atoms occupying the same site were restricted to be equal. The sums of occupancies of Mn1/Fe1 and Mn2/Fe2 were restricted to the ideal values and the overall chemical composition was restricted to Mn_3_Fe_2_Si_3_ in accordance with the result from chemical analysis (Table S1 in the supporting information).

For the single-crystal refinements, the occupancies for the Mn and Fe sites were fixed to the values obtained from refinement of the neutron powder data. Further details concerning the refinement, together with atomic coordinates and displacement parameters, are given in Tables S2–S4 in the supporting information: CCDC reference numbers 2202008–2202017.

#### 3.2.2. Temperature dependence of the crystal structure of Mn_3_Fe_2_Si_3_

Refinement of the synchrotron single-crystal diffraction data shows that Mn_3_Fe_2_Si_3_ crystallizes in the hexagonal space group *P*6_3_/*mcm* at all measured temperatures with unit-cell parameters *a* = 6.8534 (3) Å and *c* = 4.7556 (2) Å [*V* = 193.437 (15) Å^3^] at room temperature, in good agreement with the literature (Bińczycka *et al.*, 1973 ▸). Atomic positions and interatomic distances of Mn_3_Fe_2_Si_3_ refined from synchrotron single-crystal diffraction data as a function of temperature are presented in Tables S4 and S5, respectively.

To determine the distribution of the Mn and Fe atoms on the distinct crystallographic sites, a combined refinement of the synchrotron single-crystal and the room-temperature neutron powder data was performed. It shows that the *M*1 (Wyckoff position 4*d*) site in the [(*M*1)Si_6_] octahedra is occupied by 76.5 (1) at.% Fe and 23.4 (1) at.% Mn, while the *M*2 (Wyckoff position 6*g*) site is occupied by 15.6 (1) at.% Fe and 84.4 (1) at.% Mn. This preferential incorporation of Fe and Mn atoms onto the two sites is in agreement with earlier observations (Bińczycka *et al.*, 1973 ▸; Hering *et al.*, 2015 ▸).

**Figure 5:**
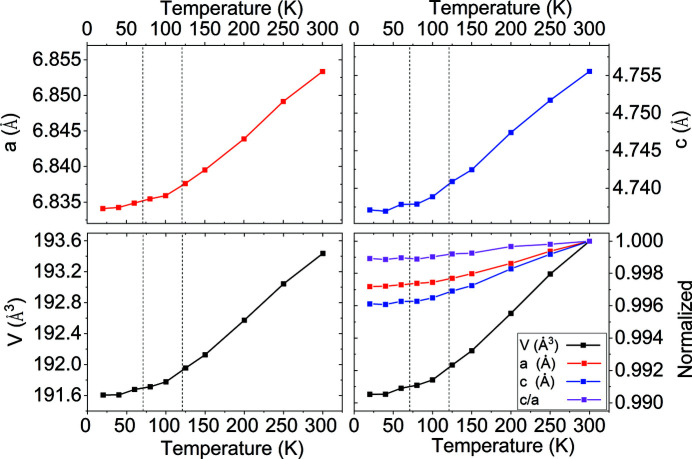
Unit-cell parameters *a* and *c* and unit-cell volume of Mn_3_Fe_2_Si_3_ as a function of temperature obtained from synchrotron X-ray single-crystal diffraction data measured during cooling. The figure at the bottom right shows the values of *a* (*b*), *c*, the *c*/*a* ratio and the unit-cell volume of Mn_3_Fe_2_Si_3_ normalized to the values at 300 K. Temperatures corresponding to the AF1–AF2 and AF2–PM transitions are indicated by dashed lines. Estimated standard deviations are smaller than the size of the symbols.

The unit-cell parameters and unit-cell volume of Mn_3_Fe_2_Si_3_ decrease smoothly as the temperature decreases, with the slope decreasing towards lower temperatures (Fig. 5 ▸; see also Table S3).

**Figure 6:**
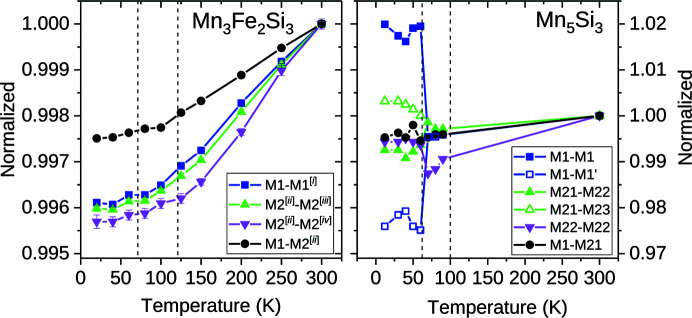
Temperature dependence of *M*–*M* interatomic distances of (left) Mn_3_Fe_2_Si_3_ and (right) Mn_5_Si_3_ as a function of temperature, normalized to the value at 300 K, obtained on the basis of refinements from synchrotron X-ray single-crystal diffraction data (Mn_3_Fe_2_Si_3_) and extracted from the literature data (Mn_5_Si_3_; Gottschilch *et al.*, 2012 ▸). Temperatures of the AF1–AF2 and AF2–PM transitions are indicated by dashed lines. The assignment of the distances is shown in Fig. S2 in the supporting information. Note that in Mn_5_Si_3_ the *M*1—*M*1 distances are split in the monoclinic AF1 phase. Estimated standard deviations are smaller than the size of the symbols, if not shown otherwise. Symmetry codes: (i) *x*, *y*, −*z* − 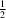 ; (ii) −*x* + 1, −*y*, −*z*; (iii) −*y* + 1, *x* − *y*, *z*; (iv) −*y* + 1, *x* − *y*, *z*.

Within the standard deviations, all interatomic distances between paramagnetic atoms in Mn_3_Fe_2_Si_3_ decrease with decreasing temperature, with no clear sign of a response to the transition from the antiferromagnetic AF1 to the antiferro­magnetic AF2 phase, or to the transition from the antiferromagnetic AF2 phase to the paramagnetic PM phase (Fig. 6 ▸; see also Fig. S2 and Table S5 in the supporting information). The interatomic distances between metal atoms located on the same sites are reduced more significantly than the distances between metal atoms located on different sites (interatomic *M*1—Si and Si—Si distances decrease in general less than *M*—*M* distances; Fig. S3).

If one compares the temperature dependence of the structure of Mn_3_Fe_2_Si_3_ with that of Mn_5_Si_3_ (Brown & Forsyth, 1995 ▸; Brown *et al.*, 1992 ▸; Gottschilch *et al.*, 2012 ▸) some major differences can be identified:

(i) While the symmetry remains hexagonal in Mn_3_Fe_2_Si_3_ over the whole investigated temperature range, *i.e.* in the stability fields of the AF2 and AF1 phases, in Mn_5_Si_3_ the PM–AF2 transition is accompanied by a reduction of the symmetry from hexagonal to orthorhombic. In addition, for Mn_5_Si_3_ a further decrease of the symmetry to monoclinic was observed by Gottschilch *et al.* (2012 ▸) for the AF1 phase, which was, however, not reported by Brown *et al.* (1992 ▸).

(ii) The *M*1—*M*1 distance in Mn_5_Si_3_ is clearly changed at the AF2–AF1 transition, while this is not observed for Mn_3_Fe_2_Si_3_. Assuming that the monoclinic model from the literature for the AF1 phase of Mn_5_Si_3_ is correct (Gottschilch *et al.*, 2012 ▸), the *M*1 position is split into two symmetrically independent positions, which in turn leads to two different *M*1—*M*1 distances parallel to **c**, one of them being significantly increased at the transition while the second one is significantly decreased (Fig. 6 ▸).

(iii) At the AF2–AF1 transition, the *M*2—*M*2 distances in Mn_5_Si_3_ clearly show a change of slope, a trend that is not observed for Mn_3_Fe_2_Si_3_.

**Figure 7:**
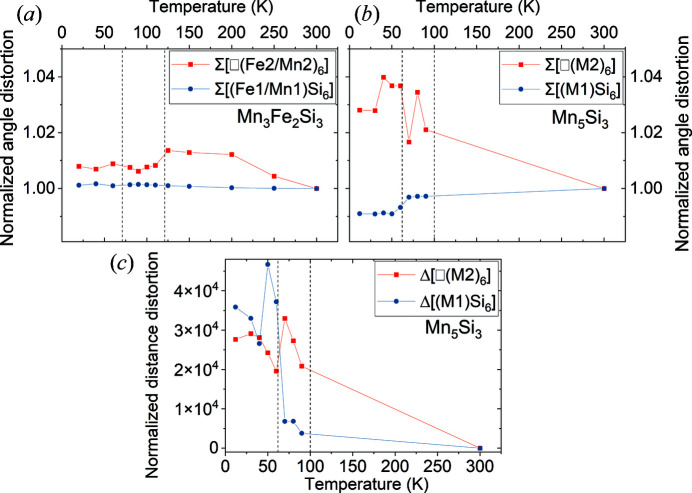
Normalized angular distortion Σ of [(*M*1)Si_6_] and [□(*M*2)_6_] octahedra as a function of temperature in (*a*) Mn_3_Fe_2_Si_3_ and (*b*) Mn_5_Si_3_ based on data from Gottschilch *et al.* (2012 ▸). (*c*) Normalized distance distortion Δ of [(*M*1)Si_6_] and [□(*M*2)_6_] octahedra as a function of temperature in Mn_5_Si_3_ based on data from Gottschilch *et al.* (2012 ▸) (values are normalized to the volumes at 300 K). Estimated standard deviations are smaller than the size of the symbols. Temperatures of the AF1–AF2 and AF2–PM transitions are indicated by dashed lines.

To compare the temperature-dependent behaviour of both compounds further, the normalized angular distortion Σ and distance distortion Δ of the [(*M*1)Si_6_] and [□(*M*2)_6_] octahedra were calculated using the program *Octadist* (Ketkaew *et al.*, 2021 ▸)^2^ and normalized to the values for 300 K (Fig. 7 ▸). It is striking that, in both compounds, the [□(*M*2)_6_] octahedra show a more pronounced distortion than the [(*M*1)Si_6_] octahedra. However, while for Mn_3_Fe_2_Si_3_ the angular distortion of the [□(*M*2)_6_] octahedra is decreased at the PM to AF2 transition and the distortion of [(*M*1)Si_6_] stays nearly constant over the whole temperature range, in Mn_5_Si_3_ the distortion of the [□(*M*2)_6_] octahedra seems to increase at the PM–AF2 transition, while the distortion of the [(*M*1)Si_6_] octahedra seems to decrease at the AF2–AF1 transition. Furthermore, in contrast to Mn_3_Fe_2_Si_3_ where all six Mn—Si distances in an octahedron are equal at all temperatures (and therefore Δ = 0), in Mn_5_Si_3_ a sudden increase in the distance distortion parameter Δ of the [□(*M*2)_6_] octahedra is observed at the temperature of the PM–AF2 transition and an additional sharp increase in the distance distortion is observed for the [(*M*1)Si_6_] octahedra at the AF2–AF1 transition (Fig. 7 ▸).

#### 3.2.3. Refinement of the magnetic structure of Mn_3_Fe_2_Si_3_

**Figure 8:**
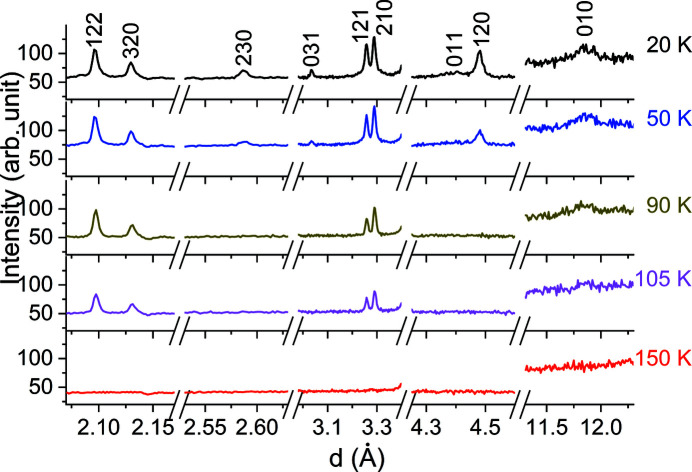
Mn_3_Fe_2_Si_3_ neutron powder diffraction data obtained on POWGEN at different temperatures for a central wavelength of 2.665 Å. The *d* regions with the strongest magnetic Bragg reflections are shown. Indices of the magnetic Bragg peaks refer to all temperatures and are based on the orthorhombic *Ccmm* setting.

The neutron powder diffraction diagrams of Mn_3_Fe_2_Si_3_ (Fig. 8 ▸) show the first peaks of magnetic origin at 105 K. At 50 K, additional weak peaks resulting from magnetic order are visible. For the derivation of the magnetic space-group symmetries the built-in algorithms of *JANA2006 ▸* (Petříček *et al.*, 2014 ▸) were used. Starting from the space group *P*6_3_/*mcm* of the nuclear structure (Tables S6 and S7) and assuming one magnetic propagation vector [*k*_hex_ = 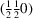 = *k*_ortho_ = (010)], eight different orthorhombic models with different magnetic moment directions are derived (Table S8).

For the refinements, the nuclear structure of Mn_3_Fe_2_Si_3_ was transformed to the orthohexagonal setting (*a*_ortho_ = *a*_hex_, *b*_ortho_ = *a*_hex_ + 2*b*_hex_, *c*_ortho_ = *c*_hex_), space group *Ccmm*.^3^ Magnetic atoms occupying the same site were restricted to have identical magnetic moments. Only magnetic moments and polynomial background parameters, which were combined with a manual background, were refined, while all other parameters were fixed to the values obtained from the refinement of the nuclear structure.

**Figure 9:**
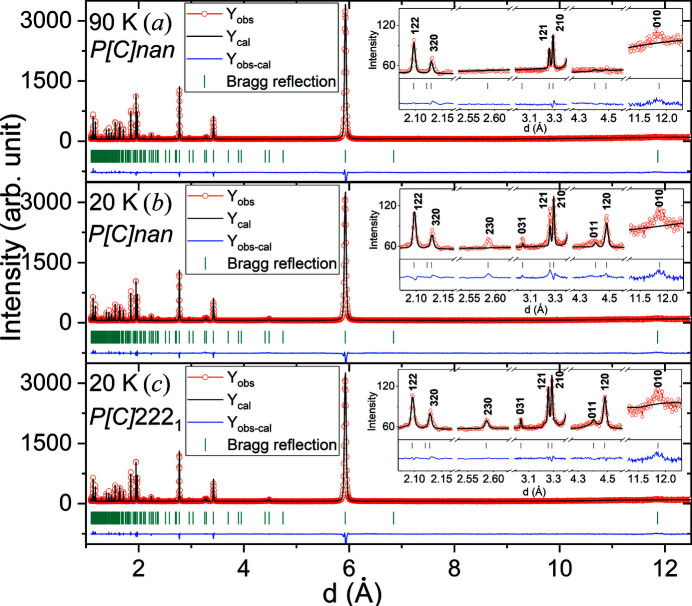
Rietveld refinements of the neutron powder data (central wavelength of 2.665 Å) of Mn_3_Fe_2_Si_3_ in magnetic space group *P*_*C*_ *nan* at (*a*) 90 K and (*b*) 20 K, and (*c*) in magnetic space group *P*_*C*_222_1_ at 20 K. Grey tick marks indicate the positions of nuclear and magnetic reflections. The difference curve is shown below. The insets present selected characteristic magnetic peaks in the 2–12.5 Å region.

From the eight refined models, the one in magnetic space group *P*_*C*_ *nan* clearly gives the best agreement factor, both at 90 and 20 K (Table S9). Figs. 9 ▸(*a*) and 9 ▸(*b*) show the corresponding Rietveld refinements.

For the data at 90 K (and 105 K), which correspond to the stability region of the AF2 phase (*T*_AF1–AF2_ ≃ 70 K < *T* < *T*_AF2–PM_ ≃ 120 K) the model in magnetic space group *P*_*C*_ *nan* fits the measured neutron diffraction pattern very well. The only exception is the 010 reflection [see inset in Fig. 9 ▸(*a*)] which is significantly broader than all other magnetic peaks and only disappears completely at 150 K.

At 20 K, that is in the stability field of the AF1 phase (*T*_AF1–AF2_ ≃ 70 K), the intensities of the newly arising magnetic peaks are underestimated in the refinement based on magnetic space group *P*_*C*_ *nan*. In particular, the 230 reflection, which is weak but clearly visible, has an intensity that is calculated to be zero in this model [see inset in Fig. 9 ▸(*b*)]. We therefore decided to lower the magnetic symmetry for the 20 K data further. For this, the nuclear structure was transformed to the different maximum *translationengleiche* subgroups of *Ccmm*, while still restricting the nuclear structure to hexagonal symmetry. For each of the different subgroups, the different magnetic models were then derived and refined. On the basis of the agreement factors (Tables S10 and S11), the model in magnetic space group *P*_*C*_222_1_ (derived from the *C*222_1_ space group) clearly appears to be the best of the 40 refined models. The Rietveld refinement of this model is shown in Fig. 9 ▸(*c*).

#### 3.2.4. Magnetic structures of Mn_3_Fe_2_Si_3_

The refinements of the magnetic structures of Mn_3_Fe_2_Si_3_ based on the neutron diffraction data at 105, 90, 50 and 20 K show two different antiferromagnetic structures: the AF2 phase corresponding to the temperature range *T*_AF1–AF2_ ≃ 70 K < *T* < *T*_AF2–PM_ ≃ 120 K and the AF1 phase corresponding to the temperature range *T* < *T*_AF1–AF2_ ≃ 70 K, in accordance with the heat capacity and magnetization data.

**Figure 10:**
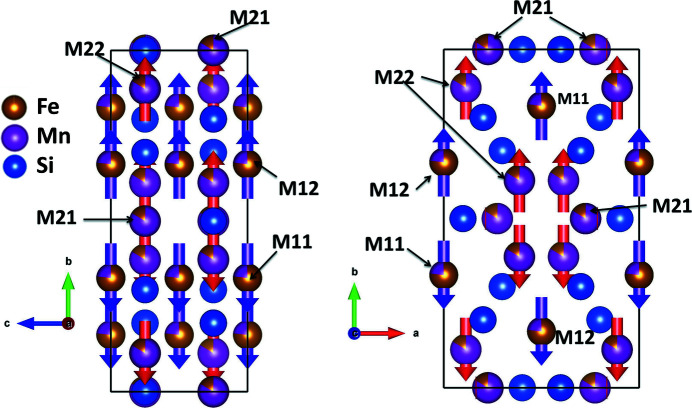
The magnetic structure of Mn_3_Fe_2_Si_3_ at 90 K (magnetic space group *P*_*C*_ *nan*). (Left) A projection along the **a** direction and (right) a projection along the **c** direction. Note that the refined value of *M*_*x*_ for Fe21/Mn21 and Fe22/Mn22 is smaller than its respective standard deviation. For the drawings, this value was therefore set to zero.

In the centrosymmetric AF2 phase (magnetic space group *P*_*C*_ *nan*), no components of magnetic moments are allowed parallel to the *c* axis (Table S8). Magnetic moments on the Mn1/Fe1 sites are aligned along the [010] direction. Moments are allowed along the [100] direction for the Fe21/Mn21 site, and in the *ab* plane for Fe22/Mn22. However, at 105 and 90 K, all refined *M*_*x*_ components for these sites are zero within error (Table 1 ▸) and consequently the AF2 structure is collinear, with all the spins aligned parallel or antiparallel to the *b* axis (Fig. 10 ▸). The Fe1/Mn1 and Fe22/Mn22 sites have refined magnetic moments of similar size, 0.70 (7) μ_B_ and 0.66 (7) μ_B_ at 105 K, respectively. When taking into account the standard deviations, the ordered moment on the Fe21/Mn21 sites is not significantly different from zero. Thus, of the Fe/Mn sites forming the empty octahedra, only two-thirds carry an ordered moment in the AF2 phase. Within these octahedra, the atoms at the same height *z* have their spins aligned in an antiparallel way. At 90 K, the observed magnetic ordering of the AF2 phase is very similar to the structure at 105 K and only the magnitude of the refined magnetic moments on Fe1/Mn1 and Fe22/Mn22 is slightly increased, with values of 0.82 (8) μ_B_ and 0.76 (8) μ_B_, respectively (Table 1 ▸).

**Figure 11:**
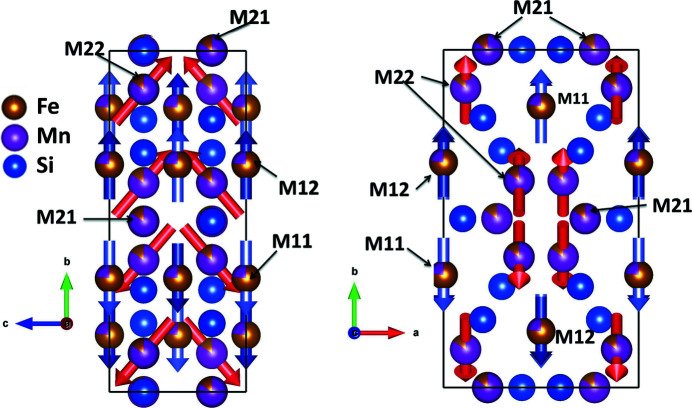
The magnetic structure of Mn_3_Fe_2_Si_3_ at 20 K (magnetic space group *P*_*C*_222_1_). (Left) A projection along the **a** direction and (right) a projection along the **c** direction. Note that the refined value of *M_x_* for Fe21/Mn21 and Fe22/Mn22 is basically identical to its standard deviation. For the drawings, this value was therefore set to zero.

At temperatures below *T*_AF1–AF2_ ≃ 70 K, the magnetic structure changes to that of the AF1 phase with symmetry *P*_*C*_222_1_ (Fig. 11 ▸). The most significant difference between the AF2 and AF1 phases is that the magnetic moments on the Fe22/Mn22 sites acquire a component in the **c** direction and align now in the *bc* plane, forming an angle of ∼30° at 50 K and ∼40° at 20 K with the *b* axis (Fig. 11 ▸). It is this re-alignment of the spins which breaks the centrosymmetry of the magnetic structure and leads to the non-collinearity of the AF1 phase. The magnitude of the Fe22/Mn22 magnetic moment is increased slightly to 0.8 (3) μ_B_ at 50 K and 1.0 (5) μ_B_ at 20 K. Moments on the *M*1 sites (now split into two magnetically independent Wyckoff positions Fe11/Mn11 and Fe12/Mn12) keep their orientation parallel to the *b* axis, and all allowed *M*_*x*_ components on the *M*21/*M*22 sites are still refined to zero within their standard deviation (Table 2 ▸).

## 4. Discussion: comparison of the magnetic structures of Mn_3_Fe_2_Si_3_ and Mn_5_Si_3_

The magnetic structure of Mn_5_Si_3_ and that of Mn_3_Fe_2_Si_3_ determined here share common features. Cooling from high temperature leads to the development of the collinear antiferromagnetic structure AF2 at 99 and 120 K, respectively, with the moments aligned parallel to the crystallographic **b** direction (referring to the orthorhombic/orthohexagonal setting).

In Mn_5_Si_3_, the PM–AF2 transition is accompanied by a structural phase transition from hexagonal to orthorhombic, while in Mn_3_Fe_2_Si_3_ the structure remains hexagonal and only the magnetic structure has a reduced symmetry. In both Mn_5_Si_3_ and Mn_3_Fe_2_Si_3_, the moments on the *M*22 site are ordered, while the Mn21 site shows no ordered moment. A major difference is the order on the *M*1 sites, which are preferentially occupied by Fe atoms in Mn_3_Fe_2_Si_3_: here they carry an ordered moment in the AF2 phase which is aligned along **b**, while in Mn_5_Si_3_ no ordered moment is observed on the *M*1 site in the AF2 phase. Upon further cooling a non-collinear structure is formed in both compounds below the temperature *T*_N1_ ≃ 70 K. The structure of this phase has been extensively discussed for Mn_5_Si_3_ in the past. The structures as described by Brown *et al.* (1992 ▸) and Gottschilch *et al.* (2012 ▸) both feature an ordered moment on the *M*1 site with a fairly large *M*_*z*_ component. In the work of Brown *et al.* (1992 ▸), moments on the *M*2 site lie mainly in the *bc* plane, while according to Gottschilch *et al.* (2012 ▸) these moments align in the *ab* plane.

In Mn_3_Fe_2_Si_3_ the moments on the *M*11/*M*12 site increase slightly and stay parallel to the **b** direction, while the moments on the *M*22 site are co-planar in the *bc* plane. Note that this structure agrees exactly with a recent proposal for the Mn_5_Si_3_ ground state based on band structure calculations (Biniskos *et al.*, 2022 ▸).

It is striking that in Mn_3_Fe_2_Si_3_ both magnetic transitions (PM–AF2 and AF2–AF1) are hardly reflected in any abrupt changes of the crystal structure {only the angular distortions of the [(*M*1)Si_6_] octahedra decrease slightly at the PM–AF2 transition}. This is significantly different in Mn_5_Si_3_ where the AF2–PM transition is combined with a change of space-group symmetry and where at the AF2–AF1 transition the inter­atomic *M*1—*M*1 distances change drastically and the octa­hedral distortions also show a pronounced change.

In earlier work it was assumed that the absence of an ordered moment on the *M*1 site in Mn_5_Si_3_ in the AF2 phase was in particular due to the Mn1—Mn1 distance being too short (2.397 Å), which was supposed to be below the critical distance that would permit magnetic ordering on the manganese (Brown & Forsyth, 1995 ▸; Shiga, 1988 ▸). The sudden increase in this distance at the AF2–AF1 transition as observed by Gourdon *et al.* (2014 ▸) (Fig. 6 ▸) would bring this distance above the critical distance, opening the possibility for an ordered moment on the *M*1 site. However, in reality the data from the literature do not provide such a uniform picture. According to Brown *et al.* (1992 ▸) and Brown & Forsyth (1995 ▸) the *M*1—*M*1 distance is 2.3967 Å in the AF2 phase at 70 K and increases slightly to 2.4021 Å at 4.2 K in AF1, so the critical distance should lie within this range. However, according to Gottschilch *et al.* (2012 ▸) things are more complicated, as here the AF2–AF1 transition is coupled to a structural transition into a monoclinic phase. In this monoclinic phase the *M*1 site splits, leading to two distinct *M*1—*M*1 distances: the first is 2.349 Å at 60 K (2.359 Å at 12 K) and the second is 2.448 Å at 60 K (2.457 Å at 12 K). So, if one assumes the monoclinic model to be correct, only half of these distances are above the critical distance. The substitution of Mn atoms with Fe atoms on the *M*1 site leads to an even smaller *M*1—*M*1 distance of 2.371 Å at the PM–AF2 transition, but ordering of the spins on these sites might well be permitted. The orbitals of the smaller Fe atoms – which predominantly occupy the *M*1 site – overlap less and hence the bandwidth is narrower, allowing the formation of an ordered moment (Shiga, 1988 ▸) already at *T*_N2_. The coupling between the magnetic structure and the crystal structure in Mn_5_Si_3_ is obvious by the strong rise in the distance distortions for the [(*M*1)Si_6_] octahedra and the formation of an ordered moment on the *M*1 sites at the AF2–AF1 transition. We argue that the site disorder in Mn_3_Fe_2_Si_3_ and the substitution of Mn atoms with the smaller Fe atoms provide additional space for the magnetic atoms, so that the magnetic order affects the crystal structure only weakly. The only pronounced effect that we observe on the crystal structure is the reduction of the angular distortions in the [□(*M*2)_6_] octahedra, which are mainly built by the larger Mn atoms.

The various magnetic features in both Mn_5_Si_3_ and Mn_3_Fe_2_Si_3_, which cannot easily be explained by the zero-field magnetic structure, require detailed single-crystal neutron diffraction in modestly strong magnetic fields of several tesla. While the magnetic response of the compounds is complex and difficult to interpret, we can state that the strongly reduced MCE in Mn_3_Fe_2_Si_3_ at the AF2–AF1 transition is related to the order on the *M*1 site, which hardly changes at this temperature. This is in excellent agreement with the recent observation that the inverse MCE associated with the AF2–AF1 transition in Mn_5_Si_3_ is due to a change in the excitation spectrum from well defined spin waves in the AF1 phase to a fluctuation-dominated excitation spectrum in the AF2 phase (Biniskos *et al.*, 2018 ▸).

## 5. Conclusion

In conclusion, we have determined the crystal and magnetic structure of Mn_3_Fe_2_Si_3_ by means of synchrotron single-crystal diffraction and neutron powder diffraction, and compared the single-crystal magnetic response of Mn_3_Fe_2_Si_3_ and the parent compound Mn_5_Si_3_. We find strong similarities in the magnetic structures with a distinct difference in the ordering on the *M*1 site. We associate these differences with the magnetocaloric properties of the compounds based on isothermal magnetization measurements, shining a spotlight on the influence of the different magnetic sites.

## Supplementary Material

Crystal structure: contains datablock(s) global, I. DOI: 10.1107/S1600576722007440/iu5027sup1.cif

Structure factors: contains datablock(s) I. DOI: 10.1107/S1600576722007440/iu5027Isup2.hkl

Additional tables and figures. DOI: 10.1107/S1600576722007440/iu5027sup3.pdf

ZIP archive of data at various temperatures. DOI: 10.1107/S1600576722007440/iu5027sup4.bin

CCDC references: 2191657, 2202008, 2202009, 2202010, 2202011, 2202012, 2202013, 2202014, 2202015, 2202016, 2202017

## Figures and Tables

**Table 1:** Refined magnetic moments of Fe/Mn atoms in the magnetic space group *P*_*C*_ *nan* for the AF2 structure of Mn_3_Fe_2_Si_3_ at 105 and 90 K *M_x_*, *M_y_* and *M_z_* are the projections of the magnetic moment along the [100], [010] and [001] directions, respectively.

Atom	*M_x_*	*M_y_*	*M_z_*
105 K			
Fe1/Mn1	−0	−0.69 (6)	0
Fe21/Mn21	−0.03 (30)	0	0
Fe22/Mn22	−0.08 (22)	−0.65 (6)	0

90 K			
Fe1/Mn1	0	−0.82 (5)	0
Fe21/Mn21	−0.01 (27)	0	0
Fe22/Mn22	−0.11 (20)	−0.75 (6)	0

**Table 2:** Refined magnetic moments of Fe/Mn atoms in the magnetic space group *P*_*C*_222_1_ for the AF1 structure of Mn_3_Fe_2_Si_3_ at 50 and 20 K *M_x_*, *M_y_* and *M_z_* are the projections of the magnetic moment along the [100], [010] and [001] directions, respectively.

Atom	*M_x_*	*M_y_*	*M_z_*
50 K			
Fe11/Mn11	0	−0.89 (7)	0
Fe12/Mn12	0	−0.79 (8)	0
Fe21/Mn21	−0.01 (60)	0	0
Fe22/Mn22	−0.13 (41)	−0.75 (2)	−0.45 (4)

20 K			
Fe11/Mn11	0	−0.89 (6)	0
Fe12/Mn12	0	−0.83 (7)	0
Fe21/Mn21	−0.03 (75)	0	0
Fe22/Mn22	−0.12 (51)	−0.78 (3)	−0.67 (3)

## Footnotes

1 The orthorhombic low-temperature phase of Mn_5_Si_3_, space group *Ccmm*, is obtained from the hexagonal phase according to the transformation *a*_ortho_ ≃ *a*_hex_, *b*_ortho_ ≃ *a*_hex_ + 2*b*_hex_, *c*_ortho_ ≃ *c*_hex_).

2 For these calculations a dummy atom at position (0, 0, 0) at the central point of the empty octahedra was introduced.

3 Note that in the orthohexagonal description, the Wyckoff position 6*g* (Fe2/Mn2) splits into two Wyckoff positions, 4*c* (Fe21/Mn21) and 8*g* (Fe22/Mn22). As the synchrotron data clearly show that the structure of Mn_3_Fe_2_Si_3_ remains hexagonal down to the lowest temperature, restrictions corresponding to the higher symmetry were applied in all refinements of the magnetic structure using local symmetry operators.
