# Supplementary material for: Site dependence of the magnetocaloric effect in Mn5−x Fe x Si3
Source: J Appl Crystallogr. 2022 Sep 6;55(Pt 5):1164–72. doi: 10.1107/S1600576722007440 (PMC9533748; doi:10.1107/S1600576722007440)
Supplement: Supplementary file 3 [file j-55-01164-sup3.pdf]

JOURNAL OF APPLIED CRYSTALLOGRAPHY

Volume 55 (2022)

Supporting information for article:

Site dependence of the magnetocaloric effect in $Mn_{5-x}Fe_xSi_3$

Mohammed Ait Haddouch, Nour Abboushi, Neetika Sharma, Andreas Eich, Andrzej Grzechnik, Cheng Li, Martin Tolkiehn, Husain Alsamamra, Jörg Voigt and Karen Friese

Site dependence of the magneto-caloric effect in $Mn_{5-x}Fe_xSi_3$: Supplementary Material

Table S.1. *Nominal composition of $Mn_3Fe_2Si_3$ as compared to results from chemical analysis (ICP-OES spectroscopy).*

Substance	Nominal Mass (g)	Nominal Mass Ratio (%)	ICP-OES results
Mn	22.85	45.69	45.6±0.3
Fe	15.48	30.96	29.3±0.2
Si	11.68	23.36	23.4±0.1

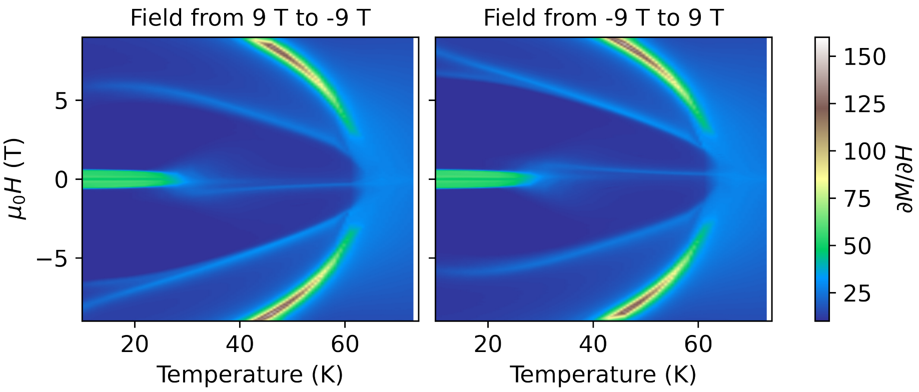

Fig. S.1. False colour plot of $\partial M/\partial H$ for an applied field $\parallel$ [001] interpolated from the isothermal magnetization curves upon field change from 9 T to -9 T (*left*) and from -9 T to 9 T (*right*) for Mn_5Si_3.

Table S.2. *Details of the structure refinements on* $Mn_3Fe_2Si_3$ *in space group* $P6_3/mcm$ *based on X-ray diffraction data. Number of variable parameters at all temperatures is 12.*

T [K]	No. of measured reflections (obs/all)	$h_{\text{min}} \longrightarrow h_{\text{max}}$ $k_{\text{min}} \longrightarrow k_{\text{max}}$ $l_{\text{min}} \longrightarrow l_{\text{max}}$	No. of symm. indep. reflections (obs/all)	R(int) obs/all	R obs/all	wR obs/all	No. of runs
300	8136/11477	-17⟶11; -18⟶14; -12⟶8	655/787	5.52/5.56	2.45/3.15	2.59/2.74	3
250	8408/11453	-17⟶11; -18⟶14; -12⟶8	668/785	5.42/5.46	2.38/2.94	2.64/2.75	3
200	8603/11415	-17⟶11; -18⟶14; -12⟶8	679/782	4.23/4.27	1.98/2.54	2.32/2.42	3
150	8601/11399	-17⟶11; -12⟶14; -12⟶8	670/779	4.14/4.18	1.98/2.51	2.22/2.32	3
125	8610/11410	-17⟶11; -18⟶14; -12⟶8	665/777	4.22/4.26	2.16/2.64	2.41/2.50	3
100	8565/11357	-17⟶11; -14⟶12; -12⟶8	662/774	4.00/4.04	2.12/2.75	2.37/2.47	3
80	8527/11378	-17⟶11; -14⟶12; -12⟶8	660/774	4.45/4.50	2.36/2.97	2.56/2.66	3
60	8449/11376	-17⟶11; -14⟶12; -12⟶8	654/774	4.40/4.46	2.35/3.01	2.57/2.70	3
40	3291/4572	-16⟶ 9; -18⟶14; -12⟶5	619/766	3.69/3.80	2.38/3.40	2.78/2.97	1
20	3220/4563	-16⟶ 9; -18⟶14; -12⟶5	617/765	3.48/3.58	2.54/3.48	2.46/2.63	1

Table S.3. *Lattice parameter and unit cell volume of $Mn_3Fe_2Si_3$ as a function of temperature.*

T (K)	a (Å)	c (Å)	V (Å^3)
300	6.85340(10)	4.75560(10)	193.441(6)
250	6.84910(10)	4.75170(10)	193.040(6)
200	6.84390(10)	4.74740(10)	192.572(6)
150	6.83950(10)	4.74250(10)	192.126(6)
125	6.83760(10)	4.74090(10)	191.955(6)
100	6.83590(10)	4.73890(10)	191.778(6)
80	6.83550(10)	4.73789(10)	191.715(6)
60	6.83490(10)	4.73790(10)	191.682(6)
40	6.8343(2)	4.73690(10)	191.608(9)
20	6.8341(2)	4.73710(10)	191.605(9)

Table S.4. *Atomic coordinates and isotropic displacement parameters of $Mn_3Fe_2Si_3$ from the refinements of x-ray single crystal diffraction data in $P6_3/mcm$. Mn1/Fe1 occupies WP 4d $(2/3, 1/3, 0)$; Mn2/Fe2 and Si occupy WP 6g $(x, 0, 1/4)$.*

T (K)	x(Mn2/Fe2)	x(Si)	U_{iso}(Mn1/Fe1)	U_{iso}(Mn2/Fe2)	U_{iso}(Si)
300	0.23281(3)	0.59802(6)	0.00619(4)	0.00788(4)	0.00692(8)
250	0.23271(3)	0.59805(6)	0.00574(4)	0.00719(4)	0.00646(8)
200	0.23259(3)	0.59805(5)	0.00481(3)	0.00596(3)	0.00551(7)
150	0.23249(3)	0.59808(5)	0.00426(3)	0.00515(3)	0.00498(7)
125	0.23247(3)	0.59806(5)	0.00388(3)	0.00465(3)	0.00457(7)
100	0.23250(3)	0.59806(5)	0.00355(3)	0.00419(3)	0.00427(7)
80	0.23246(3)	0.59809(6)	0.00359(3)	0.00417(4)	0.00432(8)
60	0.23247(3)	0.59812(6)	0.00359(4)	0.00410(4)	0.00436(8)
40	0.23246(4)	0.59807(8)	0.00380(4)	0.00416(5)	0.00442(5)
20	0.23247(3)	0.59811(7)	0.00409(4)	0.00450(4)	0.00481(9)

Table S.5. *Interatomic distances in Å of* $Mn_3Fe_2Si_3$ *in space group* $P6_3/mcm$ *obtained from synchrotron X-ray single crystal diffraction at different temperatures (The assignment of the interatomic distances is illustrated in figure S.2). Symmetry code:* $[i]x, y, -z-1/2$, $[ii]-x+1, -y, -z$, $[iii]-y+1, x-y, z$, $[iv]-y+1, x-y, z$, $[v]y+1, -x+y, -z$, $[vi]-x+1, -y, -z$, $[vii]y, -x+y+1, -z$, $[viii]-y, x-y, z$.

T (K)	M1-M1^{i} (Å)	M1-M2^{ii}/M2^{iii} (Å)	M2^{ii}-M2^{iii} (Å)	M2^{ii}-M2^{iv} (Å)	M2^{ii}-Si (Å)	M2^{ii}-Si^{v} (Å)	M2^{ii}-Si^{vi} (Å)	M1-Si^{vii} (Å)
300	2.37780(10)	2.94629(15)	2.86352(17)	2.7636(3)	2.3958(4)	2.5029(4)	2.6454(2)	2.4039(4)
250	2.37585(10)	2.94475(17)	2.86099(16)	2.7608(3)	2.3941(4)	2.5022(5)	2.6435(2)	2.4024(4)
200	2.37370(10)	2.94301(19)	2.85803(15)	2.7571(2)	2.3921(3)	2.5012(4)	2.64157(19)	2.4005(3)
150	2.37125(10)	2.94134(18)	2.85504(15)	2.7541(2)	2.3904(3)	2.5005(4)	2.63926(19)	2.3987(4)
125	2.37045(10)	2.94060(19)	2.85405(15)	2.7531(3)	2.3898(3)	2.4998(4)	2.6385(2)	2.3980(4)
100	2.36945(10)	2.93964(17)	2.85312(16)	2.7528(3)	2.3893(3)	2.4989(4)	2.6374(2)	2.3973(4)
80	2.36895(10)	2.93957(17)	2.85250(16)	2.7522(3)	2.3889(4)	2.4993(5)	2.6370(2)	2.3971(4)
60	2.36895(10)	2.93931(17)	2.85246(16)	2.7521(3)	2.3885(4)	2.4992(5)	2.6368(2)	2.3970(4)
40	2.36845(10)	2.9390(3)	2.8519(2)	2.7517(4)	2.3886(5)	2.4986(6)	2.6365(3)	2.3966(5)
20	2.36855(10)	2.9390(3)	2.85202(19)	2.7517(4)	2.3883(5)	2.4988(5)	2.6364(2)	2.39669(19)

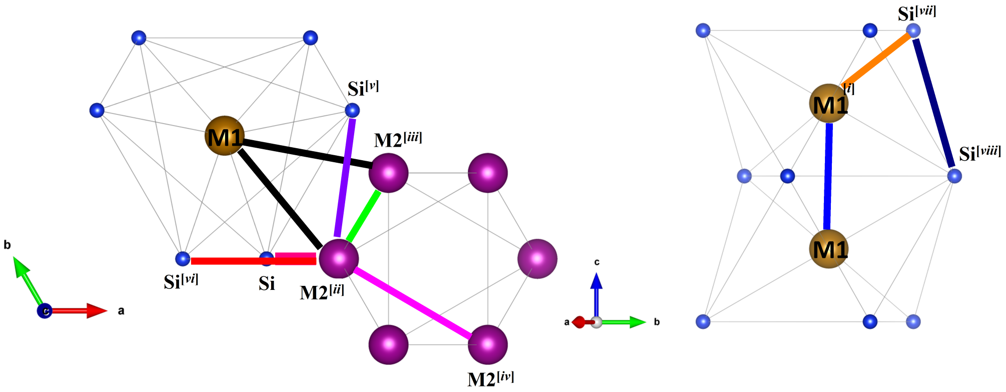

Fig. S.2. Interatomic distances in the crystal structure of $Mn_3Fe_2Si_3$. Symmetry code: $[i]x, y, -z-1/2$, $[ii]-x+1, -y, -z$, $[iii]-y+1, x-y, z$, $[iv]-y+1, x-y, z$, $[v]y+1, -x+y, -z$, $[vi]-x+1, -y, -z$, $[vii]y, -x+y+1, -z$, $[viii]-y, x-y, z$.

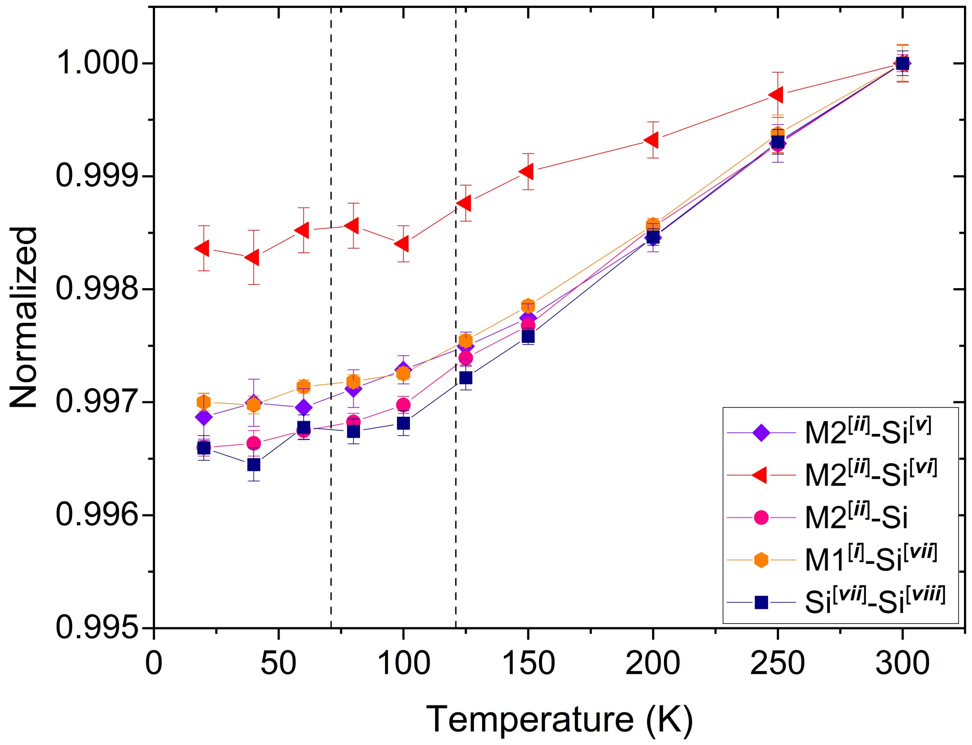

Fig. S.3. Temperature dependence of M-Si and Si-Si interatomic distances of $Mn_3Fe_2Si_3$, normalized to the value at 300 K, obtained on the basis of refinements from synchrotron X-ray single crystal diffraction data. Temperatures of the AF1-AF2 and AF2-PM transitions are indicated by dashed lines.

Table S.6. *Unit cell parameters of $Mn_3Fe_2Si_3$ described in space group $Ccmm$*

T	a	b	c
105 K	6.8429 (1)Å	11.8523Å	4.7450(1)Å
90 K	6.8421 (1)Å	11.8509Å	4.7441(1)Å
50 K	6.8405 (1)Å	11.8482Å	4.7427(1)Å
20 K	6.8395 (2)Å	11.8464Å	4.7423(1)Å

Table S.7. *Atomic coordinates and isotropic displacement parameters of* $Mn_3Fe_2Si_3$ *described in space group* $Ccmm$ *for the different temperatures. All atomic parameters are restricted to obey the higher hexagonal symmetry* $P6_3/mcm$ *(*x*=*y*(Mn22/Fe22)=0.5*x*(Mn21/Fe21) and* x*=*y*(Si2)=0.5*x*(Si1). Displacement parameters for all atoms were restricted to be equal.*

T	Mn1/Fe1			Mn21/Fe21			Mn22/Fe22			Si1			Si2			
	x	y	z	x	y	z	x	y	z	x	y	z	x	y	z	U_{iso}
105 K	1/2	−1/6	0	-0.2260(5)	0	1/4	-0.1130	x	1/4	-0.5980(2)	0	0.25	-0.2990	x	1/4	0.0024(1)
90 K	1/2	−1/6	0	-0.2258(5)	0	1/4	-0.1130	x	1/4	-0.5981(3)	0	0.25	-0.2991	x	1/4	0.0024(1)
50 K	1/2	−1/6	0	-0.2261(5)	0	1/4	-0.1130	x	1/4	-0.5979(3)	0	0.25	-0.2990	x	1/4	0.0022(1)
20 K	1/2	−1/6	0	-0.2260(5)	0	1/4	-0.1130	x	1/4	-0.5981(4)	0	0.25	-0.2991	x	1/4	0.0022(1)

Table S.8. *Derived magnetic space groups (MSG) for the magnetic structure of $Mn_3Fe_2Si_3$ based on the symmetry $Ccmm$ and the propagation vector k=(010). The allowed directions of the magnetic moments are given.*

MSG	Fe1/Mn1			Fe21/Mn21			Fe22/Mn22		
P_Ccmm	0	M_y	0	0	0	0	0	0	M_z
P_Cnam	M_x	0	M_z	0	0	M_z	0	0	M_z
P_Cnmn	0	M_y	0	0	M_y	0	M_x	M_y	0
P_Ccan	M_x	0	M_z	M_x	0	0	M_x	M_y	0
P_Cnan	0	M_y	0	M_x	0	0	M_x	M_y	0
P_Ccmn	M_x	0	M_z	0	M_y	0	M_x	M_y	0
P_Ccam	0	M_y	0	0	0	M_z	0	0	M_z
P_Cnmm	M_x	0	M_z	0	0	0	0	0	M_z

Table S.9. *R(obs/all) and wR(obs/all) factors for magnetic Bragg reflections of $Mn_3Fe_2Si_3$ based on the neutron powder data measured on POWGEN at different temperatures for a center wavelength of 2.665 Å. Magnetic space groups are derived from symmmetry Ccmm of the nuclear structure.*

	R(obs/all)							
T (K)	P_Ccmm	P_Cnam	P_Cnmn	P_Ccan	P_Cnan	P_Ccmn	P_Ccam	P_Cnmm
105	30.87/ 58.42	16.14/ 48.09	29.86/ 53.59	20.60/ 39.30	5.44/ 17.80	18.62/ 38.92	32.53/ 64.00	27.16/ 58.28
90	37.61/ 52.63	22.28/ 40.20	32.06/ 59.67	29.23/ 42.56	8.16/ 17.17	20.00/ 37.18	30.98/ 55.74	37.31/ 52.47
50	29.99/ 42.48	21.11/ 34.17	29.46/ 34.56	24.43/ 38.64	10.06/ 19.72	21.18/ 36.54	28.34/ 43.27	31.14/ 42.21
20	26.31/ 29.36	21.29/ 34.86	31.91/ 46.37	23.02/ 32.47	12.82/ 26.69	25.42/ 39.71	29.42/ 41.70	31.76/ 37.94
	wR(obs/all)							
T (K)	P_Ccmm	P_Cnam	P_Cnmn	P_Ccan	P_Cnan	P_Ccmn	P_Ccam	P_Cnmm
105	8.07/ 9.07	8.38/ 10.31	10.03/ 10.58	8.35/ 9.05	5.95/ 6.19	7.81/ 8.18	9.68/ 11.28	8.48/ 9.92
90	12.42/ 12.64	8.74/ 9.16	12.25/ 12.89	12.57/ 13.30	5.61/ 5.91	9.24/ 9.99	8.79/ 10.03	8.70/ 9.41
50	14.25/ 14.77	10.84/ 11.29	12.65/ 12.89	12.63/ 12.82	6.81/ 7.22	9.76/ 10.51	10.99/ 11.76	11.51/ 11.74
20	13.51/ 13.78	10.86/ 11.74	14.42/ 14.95	12.63/ 12.93	9.28/ 11.52	11.30/ 14.33	12.50/ 14.62	11.87/ 12.16

Table S.10. *R(obs/all) and wR(obs/all) factors for magnetic Bragg reflections of* $Mn_3Fe_2Si_3$ *based on the neutron powder data measured on POWGEN at 50 K for a center wavelength of 2.665 Å. Magnetic space groups (MSG) were derived assuming different non-isomorphic, maximal subgroups of* $Ccmm$ *as symmetry of the nuclear structure (NP: number of paremeters in the refinement).*

	$Ama2$							
MSG	P_Cc2m	P_Cn2n	P_Cc2_1n	P_Cn2_1m	P_Cc2m	P_Cn2n	P_Cc2_1n	P_Cn2_1m
R(obs/all)	27.24/31.33	9.64/13.08	23.43/27.58	21.79/28.60	25.14/30.13	9.58/13.00	22.35/26.98	21.79/28.60
wR(obs/all)	13.18/13.54	6.65/6.94	11.90/12.10	10.06/10.56	11.98/12.32	6.66/6.96	11.58/11.87	10.06/10.56
NP	6	9	11	8	6	9	11	8

	$Amm2$			
MSG	P_C2mm	P_C2an	P_C2_1am	P_C2_1mn
R(obs/all)	26.55/30.29	9.00/12.85	19.78/28.85	19.63/28.40
wR(obs/all)	13.04/13.17	6.16/6.46	9.43/10.31	9.82/10.31
NP	6	10	8	10

	$Cmc2_1$			
MSG	P_Ccm2_1	P_Cna2_1	P_Cnm2_1	P_Cca2_1
R(obs/all)	24.68/28.60	7.67/13.44	21.96/30.43	24.31/30.65
wR(obs/all)	11.57/11.82	6.24/6.44	10.29/10.69	11.60/12.10
NP	8	9	8	9

	$C222_1$			
MSG	P_C222_1	$P_C2_12_12_1$	$P_C22_12_1$	$P_C2_122_1$
R(obs/all)	7.27/12.25	21.37/28.18	24.27/26.37	21.30/29.67
wR(obs/all)	6.20/6.30	11.07/11.85	12.09/12.18	10.58/11.22
NP	7	10	9	8

	$C2/c$			
MSG	$P_C\bar{1}$	$P_C\bar{1}$	$P_C\bar{1}$	$P_C\bar{1}$
R(obs/all)	20.46/24.67	20.47/25.08	10.52/14.22	10.50/14.20
wR(obs/all)	10.84/11.13	10.94/11.25	7.05/7.33	7.05/7.32
NP	16	16	16	16

	$C2/m$			
MSG	P_C2/m	P_C2_1/a	P_C2/a	P_C2_1/m
R(obs/all)	22.66/29.49	20.83/25.15	10.98/14.83	21.67/31.41
wR(obs/all)	11.09/11.39	10.62/10.92	7.35/7.59	9.95/10.49
NP	7	10	8	9

	$C2_1/m$			
MSG	P_C2_1/m	P_C2_1/n	P_C2_1/n	P_C2_1/m
R(obs/all)	22.48/26.81	10.84/14.67	20.43/25.51	24.21/34.28
wR(obs/all)	11.41/11.71	6.99/7.27	10.75/11.06	10.26/10.70
NP	7	10	10	7

Table S.11. *R(obs/all) and wR(obs/all) factors for magnetic Bragg reflections of $Mn_3Fe_2Si_3$ based on the neutron powder data measured on POWGEN at 20 K for a center wavelength of 2.665 Å. Magnetic space groups (MSG) were derived assuming different non-isomorphic, maximal subgroups of $Ccmm$ as symmetry of the nuclear structure (NP: number of parameters in the refinement).*

	$Ama2$							
MSG	P_Cc2m	P_Cn2n	P_Cc2_1n	P_Cn2_1m	P_Cc2m	P_Cn2n	P_Cc2_1n	P_Cn2_1m
R(obs/all)	24.95/32.99	10.04/18.37	25.05/33.22	30.08/47.73	24.79/31.53	12.37/20.27	22.84/26.41	23.98/35.83
wR(obs/all)	12.68/12.97	7.55/7.75	13.45/13.75	11.91/12.76	12.23/12.39	7.86/8.03	12.66/12.81	11.09/13.35
NP	6	9	11	8	6	9	11	8

	$Amm2$			
MSG	P_C2mm	P_C2an	P_C2_1am	P_C2_1mn
R(obs/all)	29.83/34.49	8.83/16.89	26.29/34.00	21.82/31.11
wR(obs/all)	13.54/13.62	6.49/6.82	12.13/12.34	9.72/10.20
NP	6	10	8	10

	$Cmc2_1$			
MSG	P_Ccm2_1	P_Cna2_1	P_Cnm2_1	P_Cca2_1
R(obs/all)	22.79/27.72	13.23/17.35	21.25/34.93	23.97/31.42
wR(obs/all)	11.23/11.39	8.73/8.86	10.99/11.60	12.78/13.25
NP	8	9	8	9

	$C222_1$			
MSG	P_C222_1	$P_C2_12_12_1$	$P_C22_12_1$	$P_C2_122_1$
R(obs/all)	8.02/13.42	19.29/29.72	21.06/28.31	18.29/27.98
wR(obs/all)	6.91/7.01	13.14/13.32	12.61/12.78	10.71/11.31
NP	7	10	9	8

	$C2/c$			
MSG	$P_C\bar{1}$	$P_C\bar{1}$	$P_C\bar{1}$	$P_C\bar{1}$
R(obs/all)	22.16/31.17	19.85/31.69	10.62/19.67	10.77/19.23
wR(obs/all)	10.94/11.31	10.77/11.27	7.76/8.03	7.65/8.19
NP	16	16	16	16

	$C2/m$			
MSG	P_C2/m	P_C2_1/a	P_C2/a	P_C2_1/m
R(obs/all)	23.38/31.07	20.97/32.97	11.73/19.82	21.92/32.86
wR(obs/all)	10.73/11.07	11.46/12.13	8.17/8.65	10.38/10.85
NP	7	10	8	9

	$C2_1/m$			
MSG	P_C21/m	P_C2_1/n	P_C2_1/n	P_C2_1/m
R(obs/all)	26.43/34.07	8.87/18.74	21.63/27.82	41.08/53.62
wR(obs/all)	11.95/12.20	7.33/7.53	11.23/13.06	9.69/10.05
NP	7	10	10	7
